# On the Use of the Fricke-Pluronic F-127 Gel Dosimeter for Radiation Isocenter Testing of a Medical Linear Accelerator

**DOI:** 10.3390/ma17071521

**Published:** 2024-03-27

**Authors:** Michał Piotrowski, Piotr Maras, Marek Kozicki

**Affiliations:** 1 Department of Mechanical Engineering, Informatics and Chemistry of Polymer Materials, Faculty of Materials Technologies and Textile Design, Lodz University of Technology, 90-543 Lodz, Poland; michal.piotrowski@dokt.p.lodz.pl; 2 Department of Radiotherapy Planning, Copernicus Hospital, 93-513 Lodz, Poland; p.maras@kopernik.lodz.pl; 3 GeVero Co., 90-980 Lodz, Poland

**Keywords:** Fricke gel dosimeter, radiotherapy, dosimeter, radiation isocenter, polyGeVero-CT

## Abstract

This work presents a Fricke-XO-Pluronic F-127 2D radiochromic dosimeter with a flat-bed scanner for 2D reading and a dedicated data processing software package as a tool for performing coincidence testing of the radiation and mechanical isocenter of a medical accelerator. The optimal irradiation parameters were determined as follows: monitor units per beam and multi-leaf collimator gap, which are ≤750–≤2500 MU and 2–5 mm, respectively, for a cuboidal container with dimensions of 12 × 12 × 0.3 cm^3^. Despite the diffusion of Fe^3+^ ions occurring during irradiation, 2D reading can be performed at least 3 h after irradiation, without affecting the calculation performance of the coincidence test. The test was successfully performed for various irradiation settings. Overall, the Fricke-XO-Pluronic F-127 dosimeter has proven to be a potential tool for the coincidence testing of medical accelerators.

## 1. Introduction

Radiotherapy has developed significantly in recent years. More and more accurate medical accelerators and new treatment planning methods are emerging. Techniques for the dosimetry of ionizing radiation dose distribution, including gel dosimeters, are being developed simultaneously. New 3D dosimeters have been proposed and existing ones have been improved, including both polymer [1,2,3,4] and radiochromic dosimeters [5,6,7]. Recently, 4D dosimeters have also been proposed [8]. Gel dosimeters can be used not only to verify treatment plans, but also as a tool for testing the parameters of medical accelerators, such as the radiation isocenter [9,10].

The radiation isocenter is defined as the central point of the smallest sphere through which all central axes of the photon beam pass for any angular settings of the gantry, collimator, and table of the medical accelerator [11]. Determining the radiation isocenter involves calculating its radius and distance from the mechanical isocenter (intersection of laser beam position for an accelerator). This can be achieved using methods such as electronic portal imaging devices (EPIDs) [12,13], the Winston–Lutz test [14], and star shot measurements with flat 2D dosimeters [15,16,17]. The use of 3D polymer gel dosimeters such as PAGAT [10,18], NIPAM [19], PABIG^nx^ [20,21], and VIP [22] has also been proposed.

The Fricke dosimeter [23] has been one of the most important chemical dosimeters for almost a hundred years. In the 1980s, it was introduced into a gelatin matrix, which allowed for the obtention of a 3D dosimeter in which the dose distribution was read using MRI [24]. The main disadvantage of the Fricke 3D dosimeter was the high diffusion coefficient of Fe^3+^ ions, which forced the reading of the dose distribution to occur in a very short time after irradiation. One of the attempts to reduce diffusion in Fricke dosimeters was the addition of xylenol orange dye, which is able to bind to Fe^3+^ ions, creating complexes many times larger than these ions alone [25]. This approach did not significantly affect the value of the diffusion coefficient [26], but it made it possible to read the dose distribution in dosimeters using optical computed tomography (OCT) [27]. In recent years, 3D Fricke dosimeters have been proposed in various gel matrices such as gelatin [28,29,30], PVA [31,32], and Pluronic F-127 [33,34,35]. In order to reduce diffusion, a dosimeter with a gelatin matrix with the addition of sucrose was also presented [30]. Such a composition containing 40% sucrose allowed for a reduction in the diffusion coefficient of several times, but, as noted, sucrose is optically active, which may result in artifacts when reading the dose distribution from the 3D dosimeter.

The aim of this work was to examine the 2D Fricke-XO-Pluronic F-127 dosimeter as a potential tool for determining the radiation isocenter of the TrueBeam medical linear accelerator (X-rays) used in radiotherapy (Varian, Palo Alto, CA, USA). As part of the research, the number of monitor units (MU) and the dimensions of the fields that should be irradiated were optimized to obtain a result with appropriate accuracy. Sample scanning was performed over time (up to 24 h after irradiation) to check the influence of Fe^3+^ ions diffusion on the obtained results and to determine the optimal time after irradiation at which the reading should be taken.

## 2. Materials and Methods

### 2.1. Dosimeter Preparation

To prepare the Fricke-XO-Pluronic F-127 dosimeter, 25% *w*/*w* Pluronic F-127 suitable for cell culture (Pluronic^®^ F-127, Sigma-Aldrich, BioReagent, Saint Louis, MO, USA), 50 mM sulfuric acid pure per analysis (p. p. a.) (H_2_SO_4_, Chempur, Piekary Śląskie, Poland), 1 mM ferrous ammonium sulphate p. p. a. ((NH_4_)_2_Fe(SO_4_)_2_·6H_2_O, FAS, Chempur, Piekary Śląskie, Poland), and 0.165 mM xylenol orange disodium salt suitable for spectrophotometric determination of metal ions (XO, Sigma-Aldrich, Saint Louis, MO, USA) were used. First, Pluronic F-127 was dissolved in 80% of the final volume of water. To dissolve, it was added to the beaker in layers alternating with water, and then the beaker was placed in the refrigerator until the Pluronic was completely dissolved. The dosimeter components were dissolved in the remaining volume of water. The prepared solution was added to the Pluronic F-127 solution, stirring gently to avoid foaming [5]. Double distilled water was used to prepare all samples because impurities impair the stability of Fe^2+^.

### 2.2. Sample Preparation

**Figure 1:**
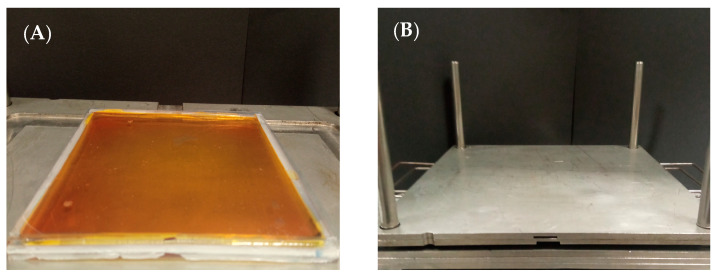
(**A**) Fricke-XO-Pluronic F-127 dosimeter in a cuboidal container, (**B**) stainless steel device to remove excess dosimeter solution by adding pressure on top of the container (the cuboidal container is between the stainless steel plates).

The 2D dosimeter was obtained by pouring the dosimeter solution into a cuboidal container (dimensions of which were 12 cm × 12 cm × 0.3 cm) made of poly(methyl methacrylate) (PMMA) (Figure 1A). During the preparation of the sample, the container was placed on a leveled stainless steel plate. Excess solution was removed by covering the container with foil and then putting another two stainless steel plates on the prepared sample (Figure 1B). Additional pressure on the container was maintained until the poured solution turned into a gel.

### 2.3. Irradiation

All samples were irradiated using a medical accelerator (TrueBeam, Varian, Palo Alto, CA, USA). In order to determine the optimal irradiation parameters, the Fricke-XO-Pluronic F-127 dosimeter samples were irradiated with different gaps of the high-definition multileaf collimator (HD MLC) of the accelerator and different monitor units (MU). One sample was irradiated with the following MU: 500, 1000, 1500, 2000, 2500, 5000, 7500, and 10,000 MU, while the gap of the HD MLC was set to 5 mm. Another sample was irradiated with 2500 MU and the following gaps of HD MLC were used: 0, 1, 2, 5, 10, and 20 mm. In each case, the samples were put on the five slabs (the thickness of each slab was 1 cm) of the SP34 RW3 phantom (IBA, Schwarzenbruck, Germany), perpendicularly to the central axis of the radiation beam. Another two slabs (1 cm and 0.5 cm thick) of the phantom were put on top of the dosimeter. Each sample was irradiated with X-rays, with a 10 MV FFF (flattening filter free) beam and monitor unit rate of 2400 MU/min, while the gantry and collimator were set to 0° and the jaw size was X: 4 cm, Y: 3 cm. The accelerator isocenter was set on the surface of the samples, with a source to surface distance (SSD) of 100 cm.

In order to determine the radiation isocenter, two samples were irradiated with a 2D star shot pattern. One of them was irradiated with 2500 MU per beam and the other with 750 MU. The remaining conditions of irradiation were as follows: X-rays, 10 MV FFF, monitor unit rate of 2400 MU/min, 5 mm gap of the HD MLC, jaw size X: 2 cm and Y: 20 cm, SSD 100 cm, collimator angle set at 0°, 90°, 150°, 240°, and gantry set to 0°. Samples were positioned between SP34 RW3 plates, in the same way as mentioned above, perpendicularly to the central axis of the radiation beam. Before irradiation, the dosimeters were placed in the isocenter of the accelerator using the LAP laser system (LAP GmbH Laser Applikationen, Lüneburg, Germany), and the accelerator isocenter was set on the surface of the samples.

### 2.4. Readout and Determining the Radiation Isocenter

The irradiated dosimeters were scanned using a flat-bed HP Scanjet G3010 scanner (Hewlett-Packard, Palo Alto, CA, USA). The scanner operated under the following settings: 75 dpi, brighten/darken option: 35 (brighten), −69 (shadows), 0 (intermediate shades), sharpen the image: none, color adjustment: none (100% saturation of colour), automatic correction of color: none. Measurements were taken within a few hours after irradiation. (i) For samples irradiated with different MUs per beam, the time equaled to 44, 109, 169, 209, 359, 419, and 1199 min; (ii) for the sample irradiated with one MU per beam and different field sizes, the time equaled to 52, 146, and 246 min; and (iii) for samples irradiated according to the 2D star shot pattern (the coincidence test), the time equaled to 40 min. During scanning, the container was covered with three sheets of white paper with a grammage of 120 g/m^2^ (POL Effect, International Paper, Kwidzyn, Poland) to properly scan the transparent gel dosimeter; otherwise, the images would contain reflections of the scanner lid. The obtained images were analyzed to determine the radiation isocenter using the polyGeVero-CT software package (v. 1.2, GeVero Co., Lodz, Poland) containing tools appropriate for processing 2D and 3D images [20,22,36].

## 3. Results and Discussion

### 3.1. Stability and Optimal MU per Field

**Figure 2:**
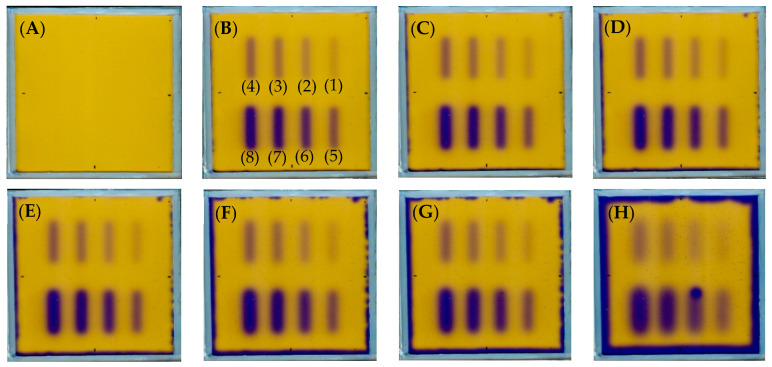
Response of the Fricke-Pluronic F-127 dosimeter to irradiation with the TrueBeam medical accelerator. (**A**) Sample just after preparation, (**B**–**H**): the same sample after irradiation (time from preparation to irradiation equals 48 min) and scanned with an HP Scanjet G3010. The time after irradiation to scanning was 44 min (**B**), 109 min (**C**), 169 min (**D**), 209 min (**E**), 359 min (**F**), 419 min (**G**), and 1199 min (**H**). Irradiation pattern: 0.5 × 4 cm^2^ stripes. The stripes irradiated with 500 (1), 1000 (2), 1500 (3), 2000 (4), 2500 (5), 5000 (6), 7500 (7), and 10,000 (8) MU, as shown in (**B**).

Fricke-XO-Pluronic F-127 was irradiated using different monitor units and gaps (HD MLC) to find the optimal irradiation parameters before assessing the accelerator radiation isocenter. The samples were scanned with an HP Scanjet G3010 scanner before and periodically within 20 h after irradiation. Images of the scanned Fricke-XO-Pluronic F-127 dosimeters are shown in Figure 2. In the irradiated areas, for each monitor unit number, the color of the sample changed from its initial orange color to purple and dark blue. Over time, the irradiated stripes became wider and blurrier due to the diffusion of Fe^3+^ ions. Moreover, due to the autoxidation of ferrous ions, the orange color of the gel on the edges of the sample changed to dark blue. The area where autoxidation occurs increased with time towards the center of the dosimeter. About 5 h after irradiation, small air bubbles appeared on the sample surface. Their number increased over time. This phenomenon is related to the construction of the cuboidal container. Due to all the changes occurring in the sample, it is suggested that the dosimeter should be scanned less than an hour after irradiation. However, as discussed later, extending the measurements to three hours after irradiation had no effect on the coincidence test calculations (see Section 3.3).

**Figure 3:**
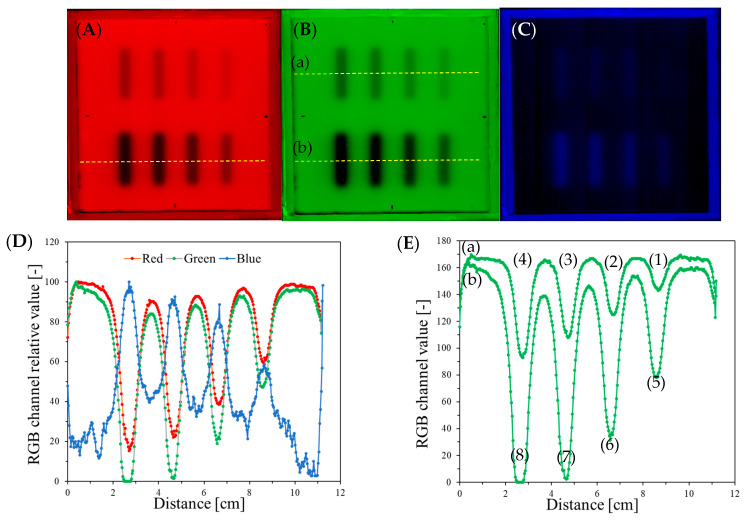
(**A**–**C**) Images correspond to the image in Figure 2B resolved into red (**A**), green (**B**), and blue (**C**) channels of RGB color model. (**D**) Profiles for red, green, and blue channels that were taken across the irradiated stripes (bottom part, as indicated in A by the yellow dashed line). (**E**) Profiles across the irradiated regions for the green channel, as indicated by the yellow dashed lines in (**B**), for top (a) and bottom (b) stripes. (1)–(8) correspond to 500, 1000, 1500, 2000, 2500, 5000, 7500, and 10,000 MU, respectively.

The images of the scanned sample were split into the red, green, and blue channels (RGB) of the RGB color model (Figure 3A–C), and the profiles (Figure 3D,E) across the irradiated areas were determined for each RGB channel to find which one contributed the most to the sample color after irradiation. The largest change was found for the green channel, which, as a consequence, was used in further data analysis.

**Figure 4:**
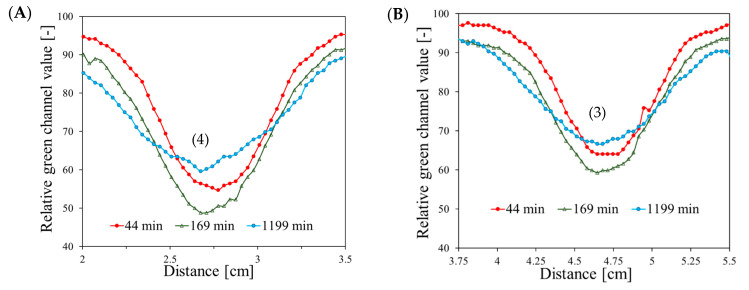

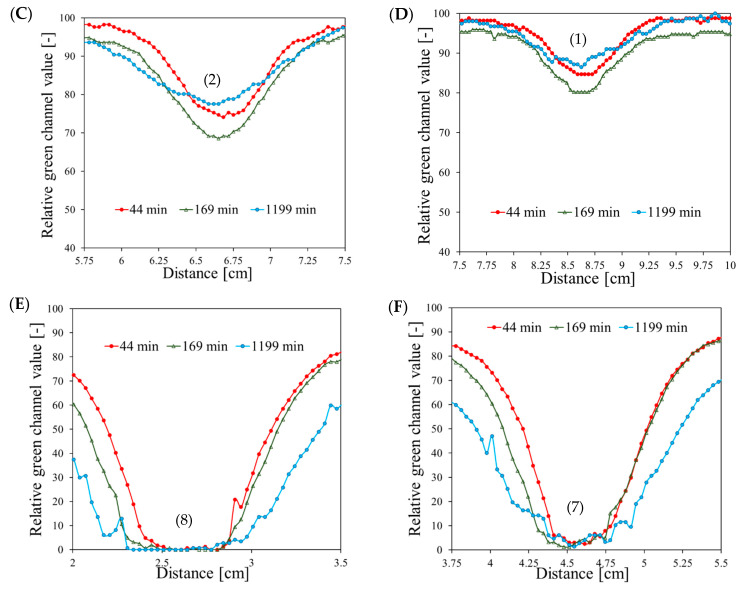

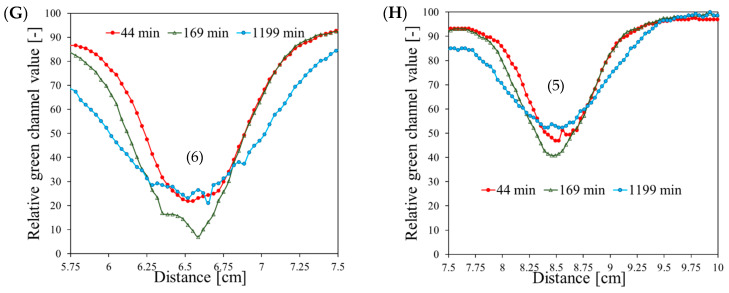
Profiles over time after irradiation of areas presented in Figure 2B–H, corresponding to top (**A**–**D**) and bottom (**E**–**H**) parts of the images in Figure 2 and their corresponding irradiated areas. (1)–(8) correspond to 500, 1000, 1500, 2000, 2500, 5000, 7500, and 10,000 MU, respectively.

Figure 4 shows the profiles across the irradiated areas 44, 169, and 1199 min after irradiation. During the time between 44 and 169 min after irradiation, the green channel value for all the irradiated areas increased. This change is related to the increase in the color intensity of the irradiated strips, indicating that the oxidation of ferrous ions in the Fricke-XO-Pluronic F-127 dosimeter occurs up to 3 h after irradiation. We suspect that the dosimeter is saturated after absorbing 10,000 MU (Figure 4E), corresponding to approximately 73.5 Gy; results reported elsewhere [33] indicate that the dosimeter should be saturated at this dose. The profiles also became wider due to the diffusion of Fe^3+^ ions in the gel matrix. The diffusion coefficient for the Fricke-XO-Pluronic F-127 dosimeter was determined by both Raman spectroscopy and optical analysis to be higher than 0 mm^2^/s (consult Table 1 in [34]) and influenced by gravity. Thus, it is possible that the results of diffusion would be clearly visible in the profiles (Figure 4) even less than three hours after irradiation. After another 1030 min, as the diffusion continued, the profiles became wider and the green channel values decreased. Where they were present, the ferric ion concentration decreased due to the increase in volume. While diffusion seems to be the limiting factor when using Fricke-XO-Pluronic F-127, the 2D scanning of samples a short time (e.g., 1–3 h) after irradiation should overcome this problem. Also, other dosimeters with, e.g., tetrazolium salts as a radiation-sensitive component, like NBT-Pluronic F-127, could be considered in the future as well, since they show no diffusion of irradiated areas over a period of storage [37].

### 3.2. Evaluation of Beam Size

**Figure 5:**
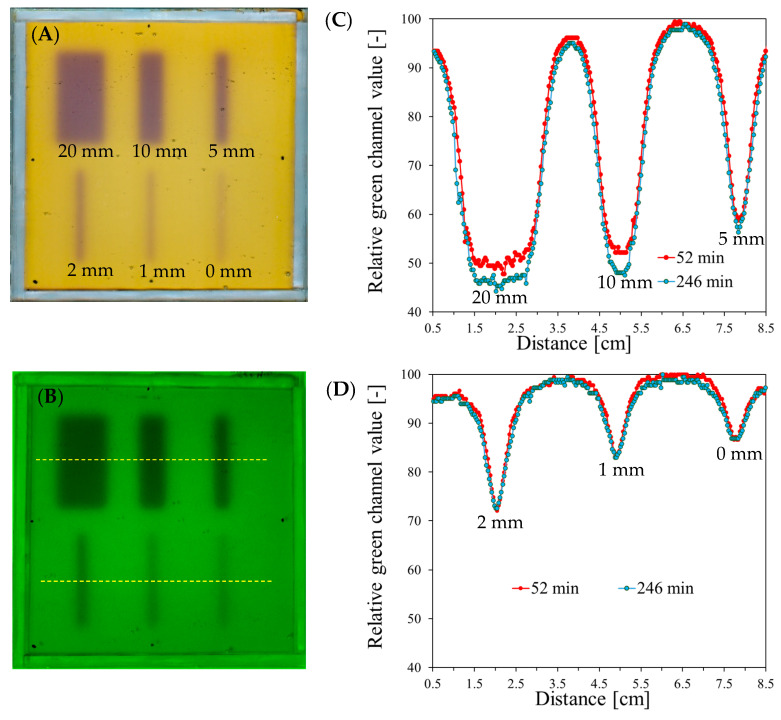
Response to irradiation of the Fricke-Pluronic F-127 dosimeter with the TrueBeam medical accelerator with 2500 MU per beam and various field sizes: 0, 1, 2, 5, 10, and 20 mm. (**A**) Image after scanning with an HP Scanjet scanner taken 52 min after irradiation. (**B**) Image of the green channel of the RGB color model; the yellow dashed lines indicate positions of the profiles across the irradiated regions presented in (**C**,**D**).

An image of the scanned sample irradiated with different field sizes (MU for every beam was 2500), the green channel images, and the profiles of the irradiated areas recorded 52 and 246 min after irradiation are presented in Figure 5.

For each field size, the change in the relative green channel value is clearly visible in the profiles. However, in the case of irradiation with 10 and 20 mm fields, there is a significant increase in the color intensiveness of the irradiated areas between 52 and 246 min after irradiation. Moreover, these areas are relatively large compared to the size of the dosimeter. For the remaining field sizes, the profiles of the irradiated areas are stable over the duration of the measurements, but due to the relatively high intensity of the color, the HD MLC gaps of 2 and 5 mm would be the most appropriate for determining the radiation isocenter.

### 3.3. Example Application: The Coincidence Test

**Figure 6:**
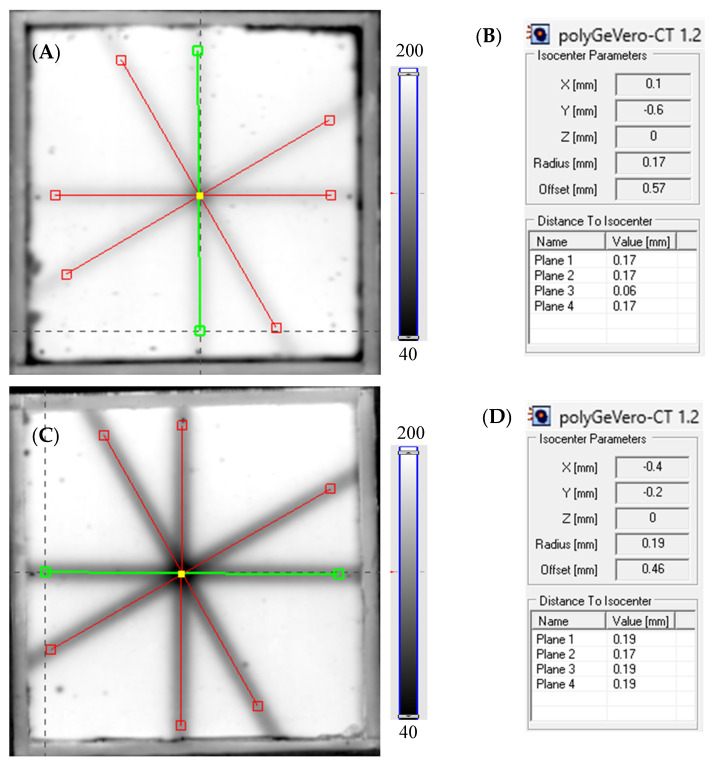
Response of the Fricke-XO-Pluronic F-127 dosimeter to irradiation with the TrueBeam medical accelerator according to the star shot pattern: the coincidence test of the mechanical and radiation isocenters. The number of monitor units per beam was 750 (**A**,**B**) and 2500 (**C**,**D**). (**A**,**C**) are images of the green channel of RGB color model ([-]) viewed in the polyGeVero-CT with tools (red and green lines) specific for the coincidence test; the yellow dot in the center of the images indicates the location of the radiation isocenter. (**B**,**D**) are the calculation results of the coincidence test.

The coincidence test of the radiation and mechanical isocenter of the TrueBeam accelerator was carried out through 2D star shot irradiation of the Fricke-XO-Pluronic F-127 dosimeters with 750 MU and 2500 MU per beam, then scanning the samples with an HP Scanjet G3010 scanner (about 40 min after irradiation) and finally calculating the isocenter parameters and MLC angles with the polyGeVero-CT software package (v. 1.2). The images of the green channel of RGB color model viewed in polyGeVero-CT for both samples are presented in Figure 6A,C and the results of the isocenter parameter calculations are presented in Figure 6B,D. The isocenter radius and offset (distance between the radiation and mechanical isocenters) are equal to 0.17 mm and 0.57 mm, and 0.19 mm and 0.46 mm for the dosimeter irradiated with 750 and 2500 MU per beam, respectively. The calculation results for both samples are within the tolerance limits for the star shot measurement: ±1 mm [38].

The results of measured MLC angles for both samples are presented in Table 1 along with the calculated differences between the planned and measured angles. For both irradiation parameters, 750 MU and 2500 MU per beam, the differences between the measured and planned angles are within the tolerance limit, which is equal to ±0.5° [39]. The obtained results clearly show that the Fricke-XO-Pluronic F-127 dosimeter in a cuboidal container is an appropriate tool for determining the radiation isocenter of the TrueBeam accelerator. High-accuracy measurements should be possible for even lower monitor units per beam settings than 750 MU, making this dosimeter a possible competitor to dosimetric films, which should be irradiated with 600 MU per beam [39]. Importantly, the Fricke-XO-Pluronic F-127 dosimeter is more ecologic. The only plastics present in this dosimeter are in the cuboidal container, which can be used multiple times. The matrix of the Fricke-XO-Pluronic F-127 dosimeter, Pluronic F-127, is also approved by the Food and Drug Administration (FDA, Silver Spring, MD, USA), as a non-toxic co-polymer [40].

**Figure 7:**
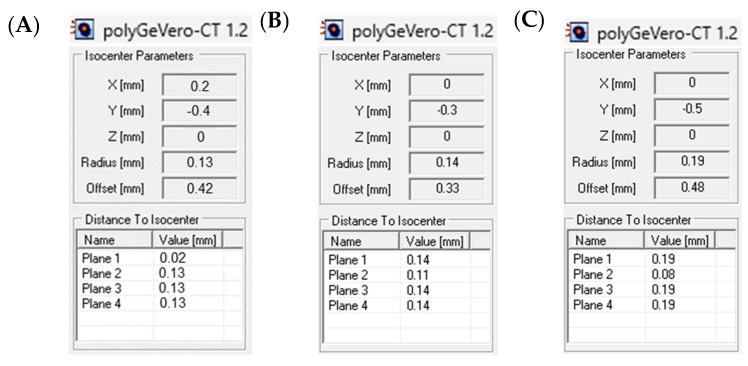
Response of the Fricke-XO-Pluronic F-127 dosimeter to irradiation with the TrueBeam medical accelerator according to the star shot pattern: the coincidence test of the mechanical and radiation isocenters. The number of monitor units per beam was 750 (**A**) and 2500 (**B**,**C**). The dosimeter was scanned after the irradiation time: 70 min after the first scanning (**A**) and 94 (**B**) and 194 min (**C**) after the first scanning (the data for the first scanning are presented in Figure 6). After scanning, the green channel images of RGB were processed in polyGeVero-CT.

Fricke-XO-Pluronic F-127 changes over time after irradiation, which is a result of the Fe^3+^ diffusion coefficient, as discussed above. In consequence, the colored stripes of the star shot irradiated dosimeter diffuse and the star image becomes blurred, analogous to the blurring of the colored stripes presented in Figure 2. This effect may deteriorate image processing towards computations related to the coincidence test. Consequently, the impact of this effect was assessed, and the results are presented in Figure 7 for the scans performed 70 min after the first scan for a dosimeter irradiated with 750 MU/beam and 94 and 194 min after the first scan for a dosimeter irradiated with 2500 MU/beam. Despite the Fe^3+^ diffusion and blurring of the images, data processing using the polyGeVero-CT software package (v. 1.2) did not pose any difficulties. The results obtained are similar to each other and to those obtained after the first scan. Otherwise, they are within the tolerance limits, just like the first scan’s results. In summary, Fricke-XO-Pluronic F-127 can be utilized for a longer period after irradiation (up to 3 h) without negatively affecting the calculations.

The system used for performing the coincidence test of the radiation and mechanical isocenter consists of a Fricke-Pluronic F-127 dosimeter in a cuboidal container made of PMMA, a flat-bed HP Scanjet G3010 scanner, and the polyGeVero-CT software package. The total uncertainty associated with such a tool results from various components: (1) positioning of the Fricke-Pluronic F-127 dosimeter container on the accelerator bench in its mechanical isocenter using the LAP laser system, which is <1 mm; (2) the precision of the data analysis performed using the polyGeVero-CT software, which is influenced by the setting of the mechanical isocenter using marks made with a pen on images of the scanned container, and is 0.34 mm; the accuracy of the defining lines along the beams in the images, which is 10^−8^ mm, and for the four lines and software algorithm, it is 8 × 10^−8^ mm; and the precision of the calculations performed using the software, which is 10^−8^. The total standard uncertainty [41] is 1.06 mm.

## 4. Conclusions

A Fricke-XO-Pluronic F-127 radiochromic 2D dosimeter in a cuboidal container made of PMMA, a flat-bed scanner, and the polyGeVero-CT software package were tested as a potential tool for performing a coincidence test of the radiation and mechanical isocenter. The optimal settings of irradiation in terms of the monitor units per beam and MLC gaps have been determined. Although the color of the dosimeter changed for each chosen monitor unit number, it is suggested to irradiate the dosimeter with <2500 MU to avoid obtaining blurred images of the irradiated areas. Each applied field size can be used to determine the radiation isocenter. For the tested cuboidal container of dimensions 12 cm × 12 cm × 0.3 cm, the optimal MLC gap is 2–5 mm. The coincidence of the radiation and mechanical isocenters were determined to be within the tolerance limit for the used medical accelerator. The differences between the measured and planned MLC angles are within the tolerance limits for both the 750 MU and 2500 MU beams. The Fricke-XO-Pluronic F-127 dosimeter is a potential competitor to the commercially available dosimetric films. The most important disadvantage of the presented dosimeter is its high diffusion coefficient, which may result in the loss of information saved in the dosimeter a few hours after irradiation. However, this can be mitigated by scanning the dosimeter shortly after irradiation, preferably within 3 h.

## Figures and Tables

**Table 1:** Results of measured MLC angles [°] and difference between planned and measured MLC angles [°] for irradiation settings with MLC angles changed during the test of coincidence of radiation and mechanical isocenter using Fricke-XO-Pluronic F-127 in a cuboidal container, an HP Scanjet 2D scanner, and data processing using the polyGeVero-CT software package. The following irradiation settings were applied: MLC gap of 5 mm, gantry set at 0°, collimator angles set at 0°, 90°, 150°, and 240°; monitor unit per beam of 750 and 2500 MU.

Planes	Planned Angles [°]	Monitor Units	
		750	2500
		Measured Angles [°]	
Plane 1	0	359.57	0.34
Plane 2	90	89.93	90.47
Plane 3	150	150.00	150.39
Plane 4	240	239.62	240.19
		**Difference between planned and measured angles [°]**	
Plane 1	0	0.43	0.34
Plane 2	90	−0.07	0.47
Plane 3	150	0	0.39
Plane 4	240	−0.38	0.19

## Data Availability

The data supporting the reported results are not stored in any publicly archived datasets. The readers can contact the corresponding author for any further clarification of the results obtained.
